# Tennessee Hospital for the Insane

**Published:** 1854-03

**Authors:** 


### TENNESSEE HOSPITAL FOR THE INSANE.

---

The Report of Dr. W. A. Cheatham to the Legislature of Tennessee on the Hospital for the Insane under his charge, is a document of great interest, and reflects credit upon his judgment, patience, and skill as Superintendent and Physician. Dr. Cheatham entered upon his duties an untried and inexperienced man, but, his report shows, not without high qualifications for the delicate and responsible office. His success has been most gratifying. No institution of the kind in our country can show better results for the last year, which is the first year of this noble charity. It is conducted upon the true principle—that of gentleness and benevolence. All that science and experience have done to ameliorate the lot of the insane in other asylums has been introduced by the enlightened Superintendent into this. He is equally fitted by nature and professional study for the management of such an institution, and under his wise administration it will prove an ornament and a blessing to the State. The situation of this Hospital is well chosen; it is in a beautiful and healthy country, a few miles from Nashville. In proof of its salubrity it is stated by Dr. Cheatham, that "for nearly a year but one death has occurred" in the Asylum.

### AMERICAN MEDICAL ASSOCIATION.

The next meeting of this National Society, our readers will please not forget, is to be held in St. Louis, in a few weeks from this time—it commences on Tuesday the 2d of May. All societies entitled to representation ought to see to the appointment of delegates who will attend the meeting. It is not to be expected that so large a number of Eastern members as have attended upon former meetings will be present at this, owing to the great travel necessary to reach the place of meeting, and for that reason there should be a much fuller attendance from the West. We trust that the strength of the western profession will be there. We owe it to ourselves to foster this Association; it is a high professional duty. If through the apathy of the members of the profession it should be allowed to languish, the whole profession would feel it.

The delegates from the University of Louisville are Prof. S. D. GROSS, and Prof. LEWIS ROGERS.

